# Immunomodulatory Role of *Ocimum gratissimum* and Ascorbic Acid against Nicotine-Induced Murine Peritoneal Macrophages *In Vitro*

**DOI:** 10.1155/2011/734319

**Published:** 2011-12-18

**Authors:** Santanu Kar Mahapatra, Subhankari Prasad Chakraborty, Somenath Roy

**Affiliations:** Immunology and Microbiology Laboratory, Department of Human Physiology with Community Health, Vidyasagar University, Midnapore 721 102, India

## Abstract

The aim of this present study was to evaluate the immune functions and immune responses in nicotine-induced (10 mM) macrophages and concurrently establish the immunomodulatory role of aqueous extract of *Ocimum gratissimum* (Ae-Og) and ascorbic acid. In this study, nitrite generations and some phenotype functions by macrophages were studied. Beside that, release of Th1 cytokines (TNF-**α**, IL-12) and Th2 cytokines (IL-10, TGF-**β**) was measured by ELISA, and the expression of these cytokines at mRNA level was analyzed by real-time PCR. Ae-Og, at a dose of 10 **μ**g/mL, significantly reduced the nicotine-induced NO generation and iNOSII expression. Similar kinds of response were observed with supplementation of ascorbic acid (0.01 mM). The administration of Ae-Og and ascorbic acid increased the decreased adherence, chemotaxis, phagocytosis, and intracellular killing of bacteria in nicotine-treated macrophages. Ae-Og and ascorbic acid were found to protect the murine peritoneal macrophages through downregulation of Th1 cytokines in nicotine-treated macrophages with concurrent activation of Th2 responses. These findings strongly enhanced our understanding of the molecular mechanism leading to nicotine-induced suppression of immune functions and provide additional rationale for application of anti-inflammatory therapeutic approaches by *O. gratissimum* and ascorbic acid for different inflammatory disease prevention and treatment during nicotine toxicity.

## 1. Introduction

Herbal remedies are demanding approach to treat several inadequate conditions of public health. In our previous lab report, aqueous extract and methanol extract of *Ocimum gratissimum* Linn protect nicotine-induced cellular damage in murine macrophages [1, 2]. But, the immunomodulatory role of aqueous extract of *O. gratissimum* against nicotine toxicity in murine macrophages has not been enlightened yet. *O. gratissimum* is an important medicinal herb, commonly known as “Ram Tulshi” in India. This plant belongs to the “*Labiaceae*” plant family member. The plant is used in folk medicine to treat different common diseases like cold and flues [3, 4]. The fresh juice of *O. gratissimum* leaves is used as a mouth antiseptic. It has been associated with chemopreventive, anticarcinogenic, free radical scavenging, radio protective, and numerous others pharmacological use [5]. The aqueous leaf extract and seed oil showed antiproliferative and chemopreventive activity on HeLa cells [6].

Ascorbic acid (AA) is used as beneficial in common medicine practice. AA plays an important role in the defense against oxidative damage, owing to its function as a reducing agent [7]. Moreover, ascorbic acid participates in the modulation of complex biochemical pathways that are an essential part of the normal metabolism of immune cell [8]. Mechanistic studies indicated that ascorbic acid can modulate the immune system by inhibiting FAS-induced monocyte apoptosis [9]. Ascorbic acid enhances the human immune response that enhances the Con A-induced proliferation of PBLs [10], chemotaxis of neutrophils [11], and phagocytosis of mononuclear leukocytes [12]. It has been reported that ascorbic acid upregulates the IgM antibody responses in a cytokine-dependent manner in spleenocytes [13]. The *in vitro* function of endotoxic shock-induced macrophages and lymphocytes was modulated by ascorbic acid [14, 15]. It also suppressed the immunobiological pathways and exerts anti-inflammatory activity in unstimulated and mitogen-stimulated peripheral blood mononuclear cells *in vitro* [16]. In a current study, Chang et al. reported that high dose of AA supplementation might attenuate allergic inflammation *in vivo* via modulating the Th1/Th2 balance toward the Th1 pole during the Th2-skewed allergic airway inflammation and decreasing eosinophilic infiltration into BALF [17]. Hence, the use of ascorbic acid (as a reference drug) to protect the adverse effect of nicotine in murine peritoneal macrophages may bring a therapeutic approach.

The inflammatory system is kept in balance through the reciprocal production of predominantly proinflammatory cytokines, including tumor necrosis factor-alpha (TNF-*α*) from T helper 1 (Th1) cells, and predominantly anti-inflammatory cytokines, including interleukin 10 (IL-10) from (primarily) T helper 2 (Th2) cells [18, 19]. This balance of pro- to anti-inflammatory cytokines is described as a ratio of Th1/Th2 type responses elsewhere [20, 21]. Macrophages are both antigen presenting cells and phagocytes and exert effects, for example, by production of cytokines like IL-12, TNF-*α*, and IFN-*γ* [22]. By secreting IL-12, they regulate the Th1/Th2 cytokine balance toward a Th1-profile. Furthermore, macrophage secretion of cytokines might provoke free radical production [23]. Th1 cells predominantly secrete TNF-*α* and IL-12, whereas Th2 cells mainly release IL-10. There are also Th3 cells, producing TGF-*β*, that act as regulators of effector T cells [24].

Nicotine, the major toxic product of cigarette smoke and other tobacco products, has been implicated in the development of free radical mediated oxidative injury in murine peritoneal macrophages, lymphocytes, and albino rat tissues [25–28]. Cigarette smoke, the main source of nicotine exposure in human health, is a major health risk factor worldwide and significantly increases the incidence of several diseases. Many of these diseases are inflammatory in nature or have an inflammatory component. Nicotine has been shown to suppress various parameters of the immune system. Downregulation of IL-10 release and mRNA expression by nicotine has been observed experimentally using a murine alveolar macrophage cell line [29], and IL-6, TNF-*α*, and IFN *γ* are released in LPS-induced murine spleenocyte [30].

In this present study, we have hypothesized that nicotine-induced alteration of the cellular functions and Th1/Th2 cytokines production in murine macrophages may be ameliorated through administration of *O. gratissimum* products. To our knowledge, there are no comprehensive studies available on this particular issue. Hence, we have approached the anti-inflammatory/beneficial role of *O. gratissimum *and ascorbic acid to restore the macrophage functions and Th1/Th2 cytokine balance in it. 

## 2. Results

### 2.1. Nitrite Generation by Macrophages

**Figure 1:**
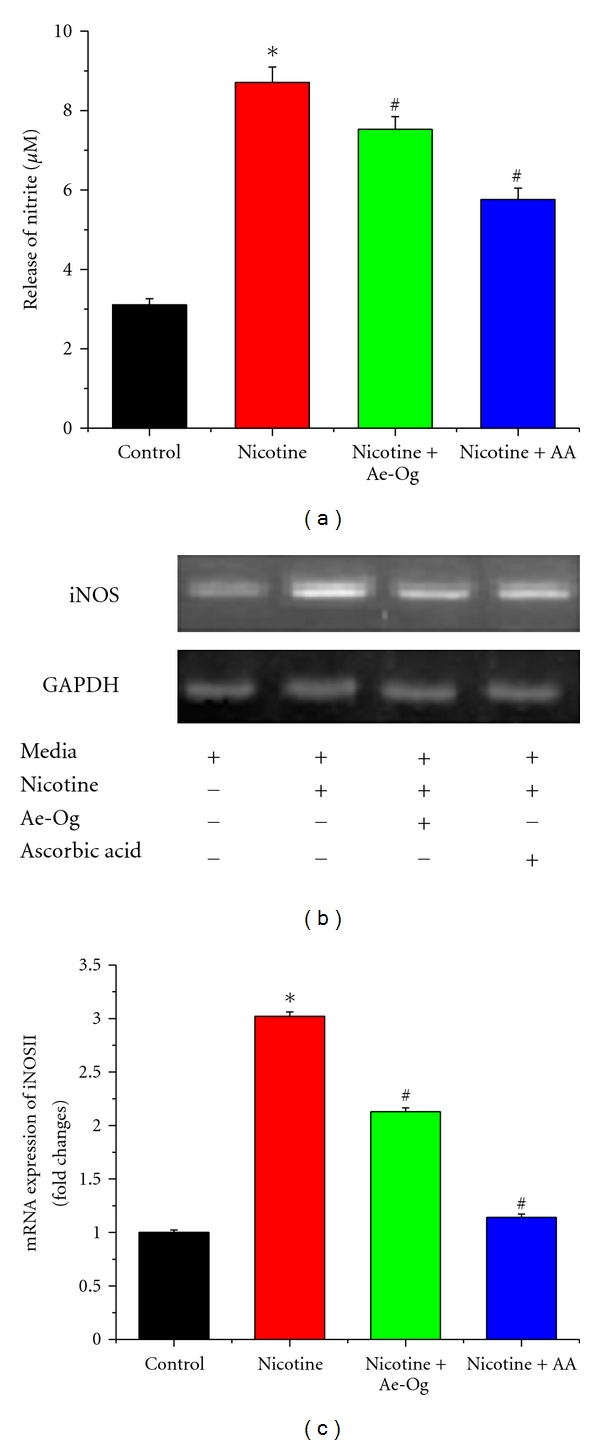
Effects of nicotine (10 mM), aqueous extract (Ae-Og) (10 *μ*g/mL), and ascorbic acid (AA) (0.01 mM) on nitrite (NO) generation and iNOSII expression in murine peritoneal macrophages are presented in Figure 1. Macrophages were treated with nicotine, nicotine + Ae-Og, and nicotine + AA. After the treatment schedule, as mentioned in Section 4, cell-free supernatants collected were subjected to the (a) nitrite generation spectrophotometrically by the Griess reaction and expressed as (*μ*M). (b) The expression levels of iNOSII mRNA transcripts in murine peritoneal macrophages were measured by quantitative real-time PCR. (c) Data, obtained from RT-PCR, are presented also as fold changes of iNOSII expression compared with normal macrophages. All the experimental results are presented as mean ± S.E.M of data obtained from three independent experiments that yielded similar results. “∗” indicates statistically significant (*P* < 0.05) induction of nitrite generation and iNOSII expression in nicotine-treated cell compared with control and “#” indicates statistically significant (*P* < 0.05) decrease of nitrite generation as well as iNOSII expression in Ae-Og and AA-supplemented macrophages compared with nicotine-treated macrophages.

We have studied the nicotine-induced nitrite generation in murine peritoneal macrophages in presence of Ae-Og/ascorbic acid by Griess reagent (Figure 1(a)). NO generation is significantly (*P* < 0.05) increased in nicotine-treated macrophage by 2.81-fold in comparison to control macrophages, which was also significantly (*P* < 0.05) ameliorated by coadministration of Ae-Og, and ascorbic acid with nicotine. To confirm the NO release, we have also studied the iNOSII expression at mRNA level after treatment schedule. We have found that iNOSII expression at the mRNA level was significantly (*P* < 0.05) increased by 3.02-fold in nicotine-treated cell, supplementation of Ae-Og and ascorbic acid can reduce the iNOSII expression significantly (*P* < 0.05) compared to nicotine-treated group and these are toward the control level (Figures 1(b) and 1(c)). Hence, Ae-Og, a naturally occurring plant product, can reduce the nitrite generation like ascorbic acid in nicotine-treated murine peritoneal macrophage.

### 2.2. Study of Macrophage Function due to Nicotine, Ae-Og, and Ascorbic Acid Treatment

**Figure 2:**
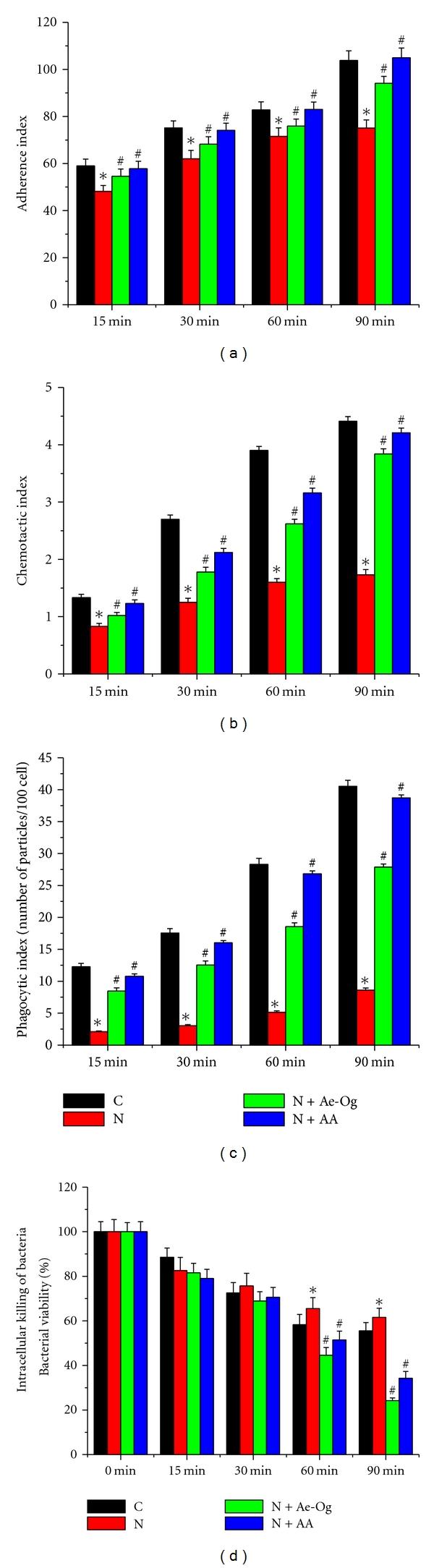
Effects of nicotine (N) (10 mM), aqueous extract (Ae-Og) (10 *μ*g/mL), and ascorbic acid (AA) (0.01 mM) on (a) adherence index, (b) chemotactic index, (c) phagocytic index, and (d) intracellular killing property of murine peritoneal macrophages are presented in Figure 2. After the treatment schedule, as mentioned in Section 4, the cells were collected and the functions of murine peritoneal macrophages were observed. Nicotine-treated cells showed the significant (*P* < 0.05) decreased functional activity in time-dependent manner, which were significantly increased during supplementation of Ae-Og and ascorbic acid. All the experimental results are presented as mean ± S.E.M of data obtained from three independent experiments that yielded similar results. “∗” indicates statistically significant (*P* < 0.05) difference of in nicotine-exposed macrophages compared with control and “#” indicates statistically significant (*P* < 0.05) difference in Ae-Og and ascorbic acid supplemented macrophages compared with nicotine-treated macrophages.

The cellular functions of peritoneal macrophages at 15, 30, 60, and 90 min after treatment schedule are shown in Figure 2. The adherence indexes (Figure 2(a)), chemotactic indexes (Figure 2(b)), phagocytosis indexes (Figure 2(c)) were increased in all groups of macrophages in time-dependent fashion. These indexes were decreased in nicotine-treated group showing statistically significant differences (*P* < 0.05) at each time interval with respect to the control group of peritoneal macrophages. But, these were increased significantly (*P* < 0.05) with supplementation of Ae-Og and ascorbic acid compared with only nicotine-treated group at each time interval. The intracellular killing of *S. aureus* at different time point by untreated and treated peritoneal macrophages was estimated to determine the intracellular killing property of macrophages (Figure 2(d)). The percentage of viable *S. aureus* at initial point was 100, which was decreased with increasing time in all types of treatment. The viability of bacteria was decreased at 15 min time point, but it was increased from 30 min to end point of the studied interval than normal macrophage. The percentages of viable bacteria were gradually decreased in normal, nicotine + Ae-Og, and nicotine + ascorbic acid-treated group at each time point of studied interval.

### 2.3. Effect of Nicotine, Ae-Og, and Ascorbic Acid on Cytokine Production

**Figure 3:**
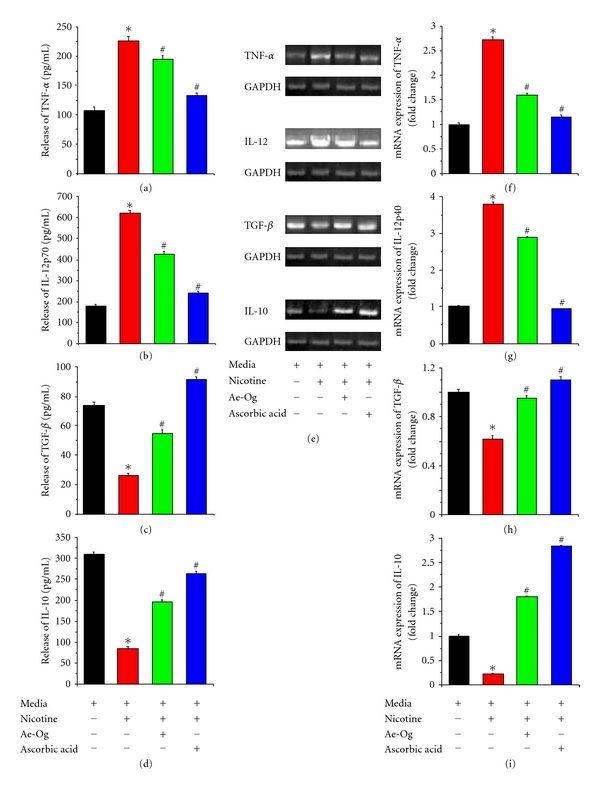
Effects of nicotine (10 mM), aqueous extract (Ae-Og) (10 *μ*g/mL), and ascorbic acid (AA) (0.01 mM) on cytokine profiles in murine peritoneal macrophages at protein and mRNA levels are presented in this figure. After the treatment schedule the cells and the cell-free supernatants were collected separately. The levels of **(**a) TNF-*α* (pg/mL), (b) IL-12p70 (pg/mL), (c) TGF-*β* (pg/mL), and (d) IL-10 (pg/mL) in the culture supernatant were evaluated by sandwich ELISA. The cells were dissolved in TRIZOL for mRNA extraction and analyzed using real-time PCR to study different proinflammatory and anti-inflammatory cytokine mRNA expressions. Quantitative real-time PCR results (e) show the expression of studied cytokines and the data are presented as fold changes compared with the control cells as follows: (f) TNF-*α*, (g) IL-12p40, (h) TGF-*β*, and (i) IL-10 mRNA. Results are expressed as means ± S.E.M. from three replicate experiments yielding similar results. “∗” indicates statistically significant (*P* < 0.05) difference of in nicotine-exposed macrophages compared with control, and “#” indicate statistically significant (*P* < 0.05) difference in Ae-Og and AA-supplemented macrophages compared with nicotine-treated macrophages.

It is well established that nicotine is an inflammatory drug that regulates the immunological functions. Hence, we planned to study whether Ae-Og and ascorbic acid treatment could modulate proinflammatory cytokine (TNF-*α* and IL-12) and anti-inflammatory cytokine (TGF-*β*, IL-10) release and expression at mRNA level in treated murine peritoneal macrophages. We observed that treatment with nicotine in macrophages showed significant increase in the release of TNF-*α* and IL-12p70 in comparison with control cells, which was significantly decreased with supplementation of Ae-Og and ascorbic acid (Figures 3(a) and 3(b)). The significant increase in TNF-*α* release might play a pivotal role in triggering the signal for enhanced NO generation. On the other hand, the release of TGF-*β* and IL-10, the signature anti-inflammatory cytokines, was significantly decreased upon nicotine treatment. Coadministration of Ae-Og and ascorbic acid with nicotine (Figures 3(c) and 3(d)) significantly increases the release of these two anti-inflammatory cytokines. To confirm our quantitative data, as observed by the ELISA method, we further studied the mRNA expression of the above cytokines after the treatment schedule by using real-time PCR analysis. The results revealed that, there was a significant increase of TNF-*α* (Figures 3(e) and 3(f)) and IL-12p40 (Figures 3(e) and 3(g)) expression in the nicotine-treated macrophages compared with control macrophages. Macrophages treated with nicotine in combination with Ae-Og/ascorbic acid showed a significant decrease in the expression levels of TNF-*α* and IL-12p40, compared with nicotine-treated macrophages. On the other hand, in nicotine-treated macrophages, there was a significant decrease of TGF-*β* (Figures 3(e) and 3(h)) and IL-10 (Figures 3(e) and 3(i)) in comparison with control macrophages at the mRNA level. When macrophages were treated with nicotine along with Ae-Og/ascorbic acid, the expression of TGF-*β* and IL-10 was significantly increased in comparison with nicotine-treated macrophages. It is the balance between the Th1 and Th2 cytokines that determines the susceptibility of different immunological disorders [31]. Our present findings showed that nicotine-treated macrophages induce Th1 cytokines and suppressed Th2 cytokines and, following treatment with Ae-Og and ascorbic acid in nicotine-treated macrophages, suppress the Th1 cytokines and enhance the Th2 cytokines, thereby preventing the vulnerability of immune disorder.

## 3. Discussion

The use of biologically active natural products is gaining increasing popularity day by day over traditional medicine as a striking alternative for the treatment of various diseases. *O. gratissimum* has been used for the medicinal purposes from the ancient ages, and recently we have found a number of beneficial roles against nicotine-induced toxicity in murine peritoneal macrophages [1, 6, 32, 33]. In this present study, we have demonstrated that *in vitro* treatment with *O. gratissimum* product (Ae-Og) significantly reduced the nicotine-induced nitrite generation in murine peritoneal macrophages (Figure 1(a)).

Peritoneal macrophages play an essential role in the immune response of the host to inflammatory and infectious processes. In the present study, we have examined some of the cellular activities of macrophages like cell adhesion, chemotactic migration, phagocytosis, intracellular killing assay, and proinflammatory and anti-inflammatory cytokine release to demonstrate the protective measures of Ae-Og and ascorbic acid against nicotine-induced modulation of cellular activities. Phagocytosis by inflammatory cells is an essential step and a part of innate immunity to protect against foreign pathogens and microorganisms or remove dead cells. The first step of phagocytosis involves adherence of phagocytic cells to tissue substrate before migration of these cells to the site of inflammation. This requires the macrophages recognition and adheres strongly enough so that they are not swept away by the flowing blood. *In vitro* cell adhesion assay may reflect the *in vivo* capacity of cellular adherence. Adhesion of macrophages shows a marked reduction in a time-dependent manner after nicotine intoxication (Figure 2(a)). Chemotactic index was significantly decreased in that of nicotine-exposed cells (Figure 2(b)). Hence it can be suggested that nicotine intoxication may somehow change the shape and functionally inactivate the macrophages, so that they migrated at a slower rate toward the chemotactic agent (fMLP). Phagocytosis is an important determinant of immune cell function. Although, free radical generation was found in higher level in nicotine-induced murine peritoneal macrophage in concentration and time-dependent manner [25, 26], in this present study, we have found that the phagocytic index and intracellular killing of bacteria were significantly decreased in nicotine-treated cell compared with control group (Figures 2(c) and 2(d)). In support of our results, many researchers had reported the decreased phagocytic and bactericidal activity by alveolar macrophages of smoker [34–36]. Besides that, in our present study, these phenotype functions (adherence capacity, chemotactic property, rate of phagocytosis, and intracellular killing) were significantly increased in time-dependent way due to supplementation of Ae-Og and ascorbic acid (Figure 2). It may be due to the effect of this bioactive principle of *O. gratissimum* and ascorbic acid to protect murine peritoneal macrophage from deleterious effect of nicotine and, simultaneously, help to restore their normal functions. So, the natural product (Ae-Og) and potent antioxidant, ascorbic acid, may be used as immune modulator drug in current future.

We have found that nicotine augments the secretion of proinflammatory cytokines and iNOSII expression in murine macrophages. The novel finding that nicotine augments proinflammatory cytokine response in macrophages may explain the molecular mechanism of nicotine-augmented immune disorder in this *in vitro* model. Nicotine may directly induce iNOSII and TNF-*α* expression in macrophages via the nicotinic acetylcholine receptors. In turn, the activated macrophages, after they infiltrate the lesion, may activate the NF-*κ*B transcriptional factor in macrophages with their secreted proinflammatory cytokines and generated oxidative stress [37]. The simultaneous increase of free radical facilitates the progression of different immune disorder. The role of NO in immune-mediated diseases is controversial [38–40]. The free radical NO directly mediates tissue destruction [38, 41], and other findings suggest a more modulatory role for NO in inflammation. Furthermore, it has been suggested that NO modulates the Th1/Th2 balance by favoring Th1 responses [39, 40]. In this study, nicotine induces NO generation (Figure 1(a)) in murine peritoneal macrophages as well as iNOSII expression in mRNA level was downregulated when the cells were supplemented with Ae-Og or ascorbic acid in presence of nicotine (Figures 1(b) and 1(c)). So, the herbal product (Ae-Og) and ascorbic acid inhibit the NO production during nicotine toxicity in murine peritoneal macrophages.

Macrophages represent the major source of cytokines; these are susceptible to regulate multiple functions of the macrophages. But, the immune regulatory role of nicotine on macrophages has not been fully clarified, and the reported data are often conflicting, especially on the effects of nicotine on cytokine production [29, 30, 42, 43]. Our finding of the toxic effect of nicotine is in agreement with the observation that nicotine can regulate the expression of chemokines like TNF-*α*, IL-12, TGF-*β*, and IL-12 (Figure 3). Therefore, nicotine alters the secretion of chemical mediators of inflammation and reduces macrophage activity [44]. It was also reported by Maes et al. that the ratio of Th1/Th2 responders has been shown to be more indicative of immune function [21]. In our present study, it is well documented that nicotine can trigger the Th1 cytokines (TNF-*α* and IL-12) and diminish the Th2 (TGF-*β* and IL-10) cytokine release (Figures 3(a)–3(d)) as well as in the mRNA level (Figures 3(e)–3(i)), indicating that nicotine may have at least short-term damaging effect on the normal immune balance. The lack of a Th2 response to counter the increased Th1 response in nicotine-exposed murine peritoneal macrophages suggests that nicotine, important ingredient of cigarette smoking, disrupts the normal healthy balance of the Th1/Th2 levels resulting in a higher vulnerability for normal cells to be at a greater risk for developing certain autoimmune disorders. These preliminary results may indicate the mechanism by which nicotine increases disease vulnerability. Beside that, we have also demonstrated the importance of Ae-Og and ascorbic acid to exert a new anti-inflammatory drug to combat against nicotine-induced immune disorder; our results clearly established that cotreatment of Ae-Og and ascorbic acid with nicotine can diminish the nicotine-induced enhanced Th1 cytokines (TNF-*α* and IL-12) release and in mRNA level as well as boost up the Th2 (IL-10 and TGF-*β*) cytokine release and mRNA level up to more or less control level (Figure 3).

In our early reports, the nicotine-induced free radical generation and oxidative damage are ameliorated by supplementation of *O. gratissimum* plant products through decreased free radical generation (1, 2, 32). It is well known that reactive oxygen species generation and its effective oxidative stress are involved in the regulation of redox-sensitive transcription factors that mediate the expression of inflammatory mediators such as cytokines and chemokines. So, in the present study, anti-inflammatory therapeutic approaches by *O. gratissimum* plant product may be directly/indirectly protecting cell from adverse effect of nicotine-induced oxidative damage in murine peritoneal macrophages by skewing the Th1 cytokines toward the Th2 cytokines.

In summary, our study has enhanced the understanding of the molecular steps leading to nicotine-induced weakening of immune functions in murine macrophages and provided additional rationale for the application of O. gratissimum plant product and ascorbic acid as anti-inflammatory therapeutic tools for different inflammatory disease prevention and treatment during nicotine toxicity.

## 4. Materials and Methods

### 4.1. Chemicals

Hydrogen tartarate salt of nicotine and sodium dodecyl sulfate (SDS) were obtained from Sigma, USA. RPMI 1640, fetal bovine serum (FBS), antibiotic solutions (cell culture grade), heparin, ethylene diamine tetra acetate (EDTA), and ascorbic acid were purchased from Himedia, India. ELISA assay Kit for tumour necrosis factor- (TNF-) *α*, interleukin- (IL-) 12p70, IL-10, and transforming growth factor- (TGF-) *β* (Quantikine M; R&D Systems, Minneapolis, MN, USA) was procured. For isolation of total RNA, TRI Reagent was purchased from Sigma, USA. dNTPs, RevertAid M-MuLV Reverse Transcriptase, oligo dT, RNase inhibitor, and other chemicals required for cDNA synthesis were purchased from Fermentas, USA. Power SYBR Green PCR Master Mix (X 2) for quantitative real-time PCR was purchased from Applied Biosystems, UK. Oligos for real-time PCR were purchased from Sigma, USA. All other chemicals were from Merck Ltd., SRL Pvt. Ltd., Mumbai, and were of the highest grade available.

### 4.2. Animals

Experiments were performed using Swiss male mice 6–8 weeks old, weighing 20–25 g. The animals were fed standard pellet diet with vitamins, and antibiotic and water were given ad libitum and housed in polypropylene cage (Tarson) in departmental animal house with 12 h light : dark cycle and the temperature of 25 ± 2°C. The animals were allowed to acclimatize for one week. The animals used did not show any sign of malignancy or other pathological processes. Animals were maintained in accordance with the guidelines of the National Institute of Nutrition, Indian Council of Medical Research, Hyderabad, India, and approved by the ethical committee of Vidyasagar University.

### 4.3. Preparation of Aqueous Extract of *Ocimum gratissimum*

*O. gratissimum *was collected from Midnapore, West Bengal, India, in September 2007, in morning. Voucher specimens were deposited at the herbarium of the Department of Botany, Vidyasagar University. The fresh aerial part of *O. gratissimum *was dried, blended, and extracted with double-distilled water (10 : 1). The mixture was filtered with Whatman filter paper (No. 1), concentrated at 38°C by a rotary evaporator, and then allowed to stand at room temperature over night. The filtration and concentration processes were repeated to yield an aqueous solution. This solution was then centrifuged at 400× g for 10 min and supernatant was freeze dried to obtain the crude aqueous extract (Ae-Og) [1].

### 4.4. Preparation of Drug

Hydrogen tartarate salt of nicotine (Sigma, USA) was dissolved in normal saline (0.9% NaCl) to get the required concentration. The pH of the nicotine solution was adjusted to 7.4 by NaOH [32]. The stock solution of Ae-Og was diluted in normal saline to get the required concentrations.

### 4.5. Isolation of the Peritoneal Macrophages and Cell Culture

All efforts were made to minimize animal suffering and to reduce the number of animals used. Macrophages were isolated by peritoneal lavage from male Swiss mice, after 24 hrs injection of 2 mL of 4% starch according to our previous report [25]. Washing the peritoneal cavity with ice cold phosphate buffer saline (PBS) supplemented with 20 U/mL heparin and 1 mM EDTA performed lavage. Care was taken not to cause internal bleeding while collecting macrophages in the exudates. The cells were then cultured in 60 mm petridishes in RPMI-1640 media supplemented with 10% FBS, 50 *μ*g/mL gentamicin, 50 *μ*g/mL penicillin, and 50 *μ*g/mL streptomycin for 24 h at 37°C in a humidified atmosphere of 5% CO_2_/95% air in CO_2_ incubator. Nonadherent cells were removed by vigorously washing three times with ice-cold PBS. Differential counts of the adherent cells used for the experiments were determined microscopically after staining with Giemsa and the cell viability evaluated by Trypan blue exclusion was never below 95%.

### 4.6. Experimental Design

The peritoneal macrophages were divided into 4 groups, containing 4 × 10^6^ cells. The cells of control and experimental groups were maintained in RPMI 1640 media supplemented with 10% FBS, 50 *μ*g/mL gentamycin, 50 *μ*g/mL penicillin, and 50 *μ*g/mL streptomycin at 37°C in a 95% air/5% CO_2_ atmosphere in CO_2_ incubator. The following groups were considered for the experiment and cultured for 12 hrs: Group I: Control (culture media); Group II: 10 mM Nicotine in culture media; and Group III: 10 mM Nicotine + 10 *μ*g Ae-Og/mL culture media; and Group IV: 10 mM Nicotine + 0.01 mM ascorbic acid in culture media. The concentration of nicotine was selected according to our previous lab report [25, 32], and the concentration of ascorbic acid and Ae-Og was followed according to our previous lab report [1].

### 4.7. Nicotine-Induced Nitrite Production (NO) in Murine Macrophage

Nitrite production by murine macrophages was measured spectrophotometrically by the Griess reaction according to our previous lab report [33]. After the treatment schedule, the cells were then centrifuged at 10,000 rpm for 20 min. The cell-free supernatants were transferred to separate microcentrifuge tube for nitric oxide release assay. To 100 *μ*L of each of the cell-free supernatant, 100 *μ*L of Griess reagent (containing 1% sulfanilamide in 5% phosphoric acid and 0.1% N-C-1 napthyl ethylene diamine dihydrochloride in 1 : 1 ratio) was added and incubated in dark at room temperature for 10 min. Reading was taken in a UV spectrophotometer at 550 nm and compared to a sodium nitrite standard curve (values ranging between 0.5 and 25 *μ*M).

### 4.8. Determination of Adherence Index (AI)

The quantification of adherence capacity was carried out by a method previously described [45]. After the experimental procedure, aliquots of 200 *μ*L of peritoneal macrophages from group were placed in eppendorf tubes and incubated at 37°C. 10 *μ*L cells were aspirated at 15, 30, 60, and 90 min interval from eppendrof tubes after gently shaking to resuspend the sedimented cells, and the number of nonadhered macrophages was determined by counting in Neubauer chambers (Blau Brand, Germany) in an optical microscope (40x magnification lens). The adherence indexes (AI) were calculated as follows: AI = 100 − [macrophages/mL supernatant)/(macrophages/mL original sample)] × 100.

### 4.9. Determination of Chemotactic Index (CI)

The chemotactic indexes of peritoneal macrophages were determined according to Wilkinson with slight modification [46]. Three small circular wells were punched out equidistantly in 0.8% agar gel. The central well was filled with peritoneal macrophages (5 × 10^4^/well). One of the peripheral wells was filled in PBS IX and the other with fMLP (10^−8^ M), a well-known chemoattractant. The chemotactic factor present in the fMLP diffuses radially through the agar and attracts the macrophages toward the fMLP. Cells were aspirated at 15, 30, 60, and 90 min from both the peripheral wells of each plate and smears were drawn separately on glass slides, allowed to air-dry, fixed in methanol, and stained with Giemsa. Chemotactic index of the respective groups was determined from the ratio of directed migration; that is, cells migrated toward the fMLP containing well to random migration; that is, cells migrated toward the PBS-containing well.

### 4.10. Determination of Phagocytic Index (PI)

Phagocytosis assay of inert particles (latex beads) was carried out following the method of De la Fuente [47]. Aliquots of 200 *μ*L of the peritoneal suspension were incubated in cell culture plates (Tarson) for 30 min. The adhered monolayer obtained was washed with prewarmed PBS, and then 200 *μ*L of Hank's medium and 20 *μ*L latex beads (diameter: 1.09 *μ*m) (Sigma) were added. After 30 min of incubation, the plates were washed, fixed, and stained, and the number of particles ingested by 100 macrophages was determined by counting in an optical microscope (100x magnification lens). The number of ingesting macrophages per 100 macrophages was also determined. 

### 4.11. Determination of Intracellular Killing Activity (IKA)

*Staphylococcus aureus* bacteria (10^7^/mL) were incubated with macrophages (10^6^/mL) in a total volume of 1 mL RPMI-BSA containing 10% normal mouse serum and this bacteria/macrophage preparation was rotated for 20 min at 37°C. Noningested bacteria were removed by differential centrifugation (10 min, 900 rpm) at 4°C and two washes with ice-cold RPMI-BSA. The cells containing ingested bacteria were resuspended in RPMI-BSA containing 10% normal mouse serum and then reincubated at 37°C (in presence of 100 *μ*g/mL gentamycin to kill extra cellular bacteria) for 1 h. Cells were washed in RPMI-BSA to remove gentamycin. After washing, cells were reincubated for 1 h and during this incubation cells were taken at different time intervals, keeping the cells in ice followed by centrifugation terminated intracellular killing. After addition of distilled water containing 0.01% BSA to the pellet, cells were disrupted by vigorously vortexing to release intracellular bacteria in the supernatant. 0.1 mL of the supernatant was serially diluted in sterile distilled water and plated onto nutrient agar to determine the number of viable intracellular bacteria. The number of colonies obtained at the beginning of the experiment (0 min after reincubation) was designated as given by 100% viable bacteria. Intracellular killing was then expressed as the percentage decrease from the initial number of viable bacteria [48].

### 4.12. Measurement of Cytokine Release by Sandwich ELISA

The level of murine TNF-*α*, IL-12p70, IL-10, and TGF-*β* in the conditioned medium of macrophage culture was measured using a sandwich ELISA Kit (Quantikine M; R&D systems). The assay was performed as per the detailed instructions of the manufacturer. The detection limit of these assays was <5.1, <2.5, <4, and <3 pg/mL for TNF-*α*, IL-12p70, IL-10, and TGF-*β*, respectively.

### 4.13. Isolation of RNA and Real-Time PCR

Total RNA was extracted from 4 × 10^6^ murine peritoneal macrophages using TRI Reagent (Sigma) according to the manufacturer's protocol. Isolated total RNA (1 *μ*g) was then reverse transcribed using Revert Aid M-MuLV Reverse Transcriptase (Fermentas, USA). The resulting cDNA was then used for real-time PCR for iNOSII and cytokines (IL-12p40, TNF-*α*, IL-10, and TGF-*β*) using an ABI 7500 real-time PCR system (Applied Biosystems, UK) with the DNA binding SYBR green dye. Glyceraldehyde-3-phosphate dehydrogenase (GAPDH) was used as a reference. The forward and reverse specific primer sequences used were as follows:

iNOSII:

forward 5′-CCCTTCCGAAGTTTCTGGCAGCAGC-3′,
reverse 5′-GGCTGTCAGAGCCTCGTGGCTTTGG-3′;

TNF-*α*:

forward 5′-GGCAGGTCTACTTTGGAGTCATTGC-3′,
reverse 5′-ACATTCGAGGCTCCAGTGAATTCGG-3′;

IL-12p40:

forward 5′-CAACATCAAGAGCAGTAGCAG-3′,
reverse 5′-TACTCCCAGCTGACCTCCAC-3′;

IL-10:

forward 5′-CGGGAAGACAATAACTG-3′
reverse 5′-CATTTCCGATAAGGCTTGG-3′;

TGF-*β*:

forward 5′-GGATACCAACTATTGCTTCAGCTCC-3′,
reverse 5′-AGGCTCCAAATATAGGGGCAGGGTC-3′;

GAPDH:

forward 5′-CAAGGCTGTGGGCAAGGTCA-3′,
reverse 5′-AGGTGGAAGAGTGGGAGTTGCTG-3′.

For real-time quantitative PCR, each reaction contained X 1 SYBR Green PCR master mixture (Power SYBR Green PCR Master Mix; Applied Biosystems, UK), 10 pmol of each primer, and 1 *μ*L of cDNA in a final volume of 20 *μ*L. The reaction conditions were as follows: initial activation step (5 min at 95°C) and cycling step (denaturation for 30 s at 94°C, annealing for 30 s at 58°C and then extension for 1 min at 72°C × 40 cycles) followed by melt curve analysis. Detection of the dequenched probe, calculation of threshold cycles (Ct values), and further analysis of these data were performed by the Sequence Detector software. Relative changes in iNOSII and cytokine (IL-12p40, TNF-*α*, IL-10, and TGF-*β*) mRNA expression were compared with an unstimulated control, normalized to GAPDH, and were quantified by the 22^−ddCt^ method. Thus, all the values for experimental samples were expressed as fold differences between the sample mRNA and the calibrator (GAPDH) mRNA. The data are presented as the mean ± S.E.M. of data from three independent experiments that yielded similar results.

### 4.14. Densitometric Analysis

Gene expression data were analyzed using a model GS-700 imaging densitometer and Molecular Analyst version 1.5 software (Bio-Rad Laboratories, Hercules, CA, USA).

## 5. Statistical Analysis

The experiments were performed three times and the data are presented as mean ± S.E.M. Comparisons of the means of control and experimental groups were made by two-way ANOVA test (using a statistical package, Origin 6.1, Northampton, MA 01060 USA) with multiple comparison *t*-tests, *P* < 0.05 as a limit of significance.
